# A recoverable AMBTC authentication scheme using similarity embedding strategy

**DOI:** 10.1371/journal.pone.0212802

**Published:** 2019-02-27

**Authors:** Wien Hong, Xiaoyu Zhou, Der-Chyuan Lou

**Affiliations:** 1 School of Electrical and Computer Engineering, Nanfang College of Sun Yat-Sen University, Guangzhou, China; 2 Department of Computer Science and Information Engineering, Chang Gung University, Taoyuan, Taiwan; 3 Stroke Center and Department of Neurology, Chang Gung Memorial Hospital, Linkou, New Taipei, Taiwan; Wuhan University, CHINA

## Abstract

In this paper, we propose an efficient method for authenticating the absolute moment block truncation coding (AMBTC) compressed images with the capability to recover tampered blocks. The existing methods may not be able to detect some types of intentional tampering. Meanwhile, the tampered blocks are only recovered by their means, causing an unpleasant mosaic-like appearance. The proposed method classifies image blocks into groups according to their similarities, and the group information is recorded for the recovery purpose. The multiple copies of the group information are scrambled and embedded into the bitmap of smooth blocks. The dominant portions of quantization levels are adjusted to generate a set of authentication code candidates. The codes with the minimal distortion are embedded into the least significant bits (LSBs) of the quantization levels. The tampered blocks can be recovered by averaging those untampered ones with the same index. The experimental results show that the proposed method not only achieves an excellent marked image quality and detectability, but also offers a satisfactory recovered result.

## Introduction

The rapid development of computing and software technologies has made people easy to enhance their photo qualities, create collages, and perform many other photo editing applications. In contrast, these modern techniques can also be used to tamper with digital images maliciously. Therefore, the development of image authentication schemes for protecting the integrity of digital images has been increasingly becoming important. In general, image authentication schemes can be classified into the signature-based [1] and fragile watermark-based [2–3] approaches. The signature-based authentication approaches obtain the signatures from images and store the obtained signatures in a trusted third party. To authenticate the image, the signatures stored in the trusted third party are extracted to verify the integrity of the image. The fragile watermark-based approaches embed fragile watermarks into the image to construct a marked one. Since the embedded watermarks are fragile, tampering of a marked image will damage the embedded watermarks. Therefore, the presence of the tampering can be detected.

Fragile watermark-based authentication approaches can be applied to the images of spatial or compressed domains. The spatial domain approaches embed authentication codes into cover images by altering pixel values directly. These approaches often use data embedding techniques [4–8] to achieve the goal of embedment. For example, Hsu and Tu [9] present an image authentication method by embedding authentication information into the least significant bits (LSBs) of image blocks. Their method is capable of detecting and locating tampered blocks of the spatial domain. Ullah et al. [10] propose a dual-purpose fragile watermark-based authentication method in which the located tampered area can be recovered. Hsu and Tu [11] extend the work of [9] and design an adaptive embedding rule for image tamper detection and recovery. Qin et al. [12] employ an overlapping embedding strategy to present a novel authentication method where the tampered blocks can be recovered pixel-wisely. Lo and Hu [13] propose a reversible authentication method based on residual histogram shifting. The tampered blocks can be successfully located, and the original image can be precisely reconstructed if none of the blocks are tampered. Hong et al. [14] improve the work presented in [13] and propose an efficient reversible authentication method using the pixel value ordering strategy.

In contrast, the compressed domain authentication approaches embed authentication codes into the images of compressed formats by altering the coefficients of compressed codes. Several authentication approaches of compressed domain such as Joint Photographic Experts Group (JPEG) [15], vector quantization (VQ) [16–17] and absolute moment block truncation coding (AMBTC) [18–24] have been proposed in the past decades. The AMBTC compresses an image block into a pair of quantization levels and a bitmap. Since the calculation of AMBTC compressed codes requires insignificant computation cost and provides acceptable image quality, it has attracted a number of researchers to investigate the AMBTC-based authentication methods. Nguyen et al. [19] design an image authentication scheme aiming at tamper detection for AMBTC compressed images. They utilize a reference table to embed authentication codes and achieve a high-quality embedded image while the capability of tamper detection is confirmed. Li et al. [20] present the other authentication method based on the AMBTC, in which the authentication codes are embedded into the quantization levels with the aid of a specially designed matrix. Chen et al. [21] recognize that the bitmap of each block is unprotected in [20], and thus propose an improved method to overcome the weakness of [20]. Hong et al. [22] propose an efficient authentication method by embedding the selected authentication codes into the quantization levels. Hong et al.’s method achieves a high marked image quality and is able to detect a variety of tampering.

While the aforementioned methods focus on authenticating the AMBTC codes, Hu et al. [25] propose an interesting authentication method which offers the capability to recover tampered blocks. Hu et al.’s method utilizes the differences of quantization levels to embed authentication codes, and uses the bitmaps of smooth blocks to carry recovery codes. Once the tampered blocks are detected, the recovery codes are extracted to recover the tampered blocks approximately. Hu et al.’s method achieves a satisfactory performance; however, the distortion introduced in their method is significant, and some of the modified quantization levels can possibly overflow or underflow. Moreover, since the embedded recovery codes are the means of blocks, apparent mosaic-like patterns can be seen in the recovered regions.

The proposed method classifies the AMBTC image blocks into groups of a similar pattern. The group information of each block, which is used to recover the tampered blocks, is scrambled and embedded into the bitmap of smooth blocks. A set of authentication codes is generated by altering the dominant portions of quantization levels, and an evaluation mechanism is employed to select the best candidate for embedment. The tampered blocks are recovered by averaging the pixel values of untampered ones of the same group. To better present the proposed method, the main symbols used in this paper and their definitions are listed in Table 1.

**Table 1:** Symbols used in this paper and their definitions.

Symbols	Definitions
*O*	Original image
*I*	The AMBTC compressed image
$\tilde{I}$	To-be-authenticated image
*a*	Lower quantization level
*b*	Higher quantization level
*B*	Bitmap
*n*×*n*	Size of image block
*m*	Averaged pixel value of a block
*ac*	Authentication codes
*w*	Length of authentication codes
*τ*	Copies of the recovery codes
*ψ*	Length of recovery codes
*T*	Predefined threshold
*N*	Total number of blocks

The rest of this paper is organized as follows. Section 2 introduces the AMBTC compression technique and Hu et al.’s method. Section 3 presents the proposed work, while Section 4 gives the experimental results. The concluding remarks are addressed in Section 5.

## Related works

We briefly introduce the AMBTC compression technique in this section. Hu et al.’s method, which will be extensively compared with the proposed method, is also presented in this section.

### The AMBTC compression technique

In 1984, Lema and Mitchell [26] developed the AMBTC compression technique, which was a variant of the technique developed by Delp and Mitchell [27]. To compress an image *O* using AMBTC, *O* is partitioned into blocks ${\left\{O_{i}\right\}}_{i=1}^{N}$ of size *n*×*n*, where *N* is the total number of blocks. For each block *O*_*i*_, the mean value $m_{i}=\frac{1}{n\times n}\sum_{j=1}^{n\times n}O_{i,j}$ is calculated, where *O*_*i*,*j*_ represents the *j*-th pixel of *O*_*i*_. The lower quantization level *a*_*i*_ is calculated by averaging the pixels in *O*_*i*_ satisfying *O*_*i*,*j*_≤*m*_*i*_. Similarly, the higher quantization level *b*_*i*_ is obtained by averaging the pixels in *O*_*i*_ satisfying *O*_*i*,*j*_>*m*_*i*_. The *j*-th bit *B*_*i*,*j*_ of bitmap *B*_*i*_ is 0_2_ if *O*_*i*,*j*_≤*m*_*i*_; otherwise, *B*_*i*,*j*_ is 1_2_. Therefore, the AMBTC code of block *O*_*i*_ can be written as a three-tuple (*a*_*i*_,*b*_*i*_,*B*_*i*_). Each block is compressed using the same procedure, and the AMBTC codes ${\left\{(a_{i},b_{i},B_{i})\right\}}_{i=1}^{N}$ of the image *O* are obtained. The decompression of the AMBTC codes is quite straightforward. We denote by *I*_*i*_ = (*a*_*i*_,*b*_*i*_,*B*_*i*_) the image block *I*_*i*_ decompressed from the tuple (*a*_*i*_,*b*_*i*_,*B*_*i*_). Let *I*_*i*,*j*_ be the *j*-th pixel of *I*_*i*_. If *B*_*i*,*j*_ = 0, *I*_*i*,*j*_ = *a*_*i*_ is set; otherwise, *I*_*i*,*j*_ = *b*_*i*_.

Here is a simple example to illustrate the AMBTC compression technique. Let *O*_*i*_ = [45,47,42,32;38,65,34,56;55,43,41,49;55,61,77,68] be the block to be compressed. The averaged value of *O*_*i*_ is *m*_*i*_ = 50.50. Therefore, *a*_*i*_ = 41, *b*_*i*_ = 62, and *B*_*i*_ = [0000;0101;1000;1111] are obtained. The decompressed block *I*_*i*_ = [41,41,41,41;41,62,41,62;62,41,41,41;62,62,62,62] can be simply obtained.

### Hu et al.’s method

In 2017, Hu et al. [25] proposed an AMBTC-based authentication scheme, which was able to recover the tampered image blocks approximately. In Hu et al.’s method, the higher and lower quantization levels are used to embed *w*-bit authentication codes, and the bitmaps of smooth blocks are used to carry the recovery information. Let *I*_*i*_ = (*a*_*i*_,*b*_*i*_,*B*_*i*_) be the *i*-th image block. The difference *d*_*i*_ = *b*_*i*_−*a*_*i*_ is calculated firstly. If *d*_*i*_≤*T*, *I*_*i*_ is classified into a smooth block, where *T* is a predefined threshold. In contrast, if *d*_*i*_>*T*, *I*_*i*_ is a complex block. The *w*-bit authentication code $ac_{i}^{w}$ is generated by $ac_{i}^{w}=rv_{i}\mathrm{mod}2^{w}$, where *rv*_*i*_ is a random integer generated from a pseudo-random number generator (PRNG). To embed $ac_{i}^{w}$ into *a*_*i*_ and *b*_*i*_, the first candidate of the higher quantization level $b_{i,1}^{\prime }=a_{i}+d_{i}-p_{i}+ac_{w}$ for replacing *b*_*i*_ is calculated, where

$$p_{i}=d_{i}\mathrm{mod}2^{w}$$ (1)

is the *w*-bit parity value. The second candidate $b_{i,2}^{\prime }$ is calculated using the following rules. If $b_{i,1}^{\prime }\geq b_{i}$, $b_{i,2}^{\prime }=b_{i,1}^{\prime }-2^{w}$. Otherwise, if $b_{i,1}^{\prime }<b_{i}$, $b_{i,2}^{\prime }=b_{i,1}^{\prime }+2^{w}$. Secondly, the candidate that is close to *b*_*i*_ is selected to replace *b*_*i*_. The selected candidate is denoted by $b_{i}^{s}$. However, the smoothness classification of the block *I*_*i*_ using the new difference $b_{i}^{s}-a_{i}$ could be changed. Hu et al. adopt a modification rule to recursively modify $b_{i}^{s}$ to $b_{i}^{*}$ such that $d_{i}^{*}=b_{i}^{*}-a_{i}$ and *d*_*i*_ have the same smoothness classification. Finally, the marked higher and lower quantization levels can be calculated by ${\hat{a}}_{i}=a_{i}+\left\lfloor (b_{i}-b_{i}^{*})/2\right\rfloor$ and ${\hat{b}}_{i}=b_{i}^{*}+\left\lfloor (b_{i}-b_{i}^{*})/2\right\rfloor$, respectively, where ⌊•⌋ denotes the floor operator.

Hu et al. use the mean values ${\left\{m_{i}\right\}}_{i=1}^{N}$ of blocks ${\left\{I_{i}\right\}}_{i=1}^{N}$ as the recovery information, and *τ* copies of them are scrambled using a key to obtain *τ* copies of scrambled mean values ${\left\{m_{i}^{\left(k\right)}\right\}}_{i=1}^{N}$, 1≤*k*≤*τ*. To embed the recovery information, the differences ${\left\{d_{i}\right\}}_{i=1}^{N}$ are sequentially visited. If *d*_*i*_≤*T*, because $0\leq m_{i}^{\left(k\right)}\leq 255$, $\{m_{i}^{\left(k\right)}{\}}_{k=1}^{\tau }$ is converted into (8×*τ*)-bit binary bitstream ${\left\{b_{r}\right\}}_{r=1}^{8\times \tau }$ [25], and the bitmap *B*_*i*_ is then replaced by ${\left\{b_{r}\right\}}_{r=1}^{8\times \tau }$. If *d*_*i*_>*T*, the bitmap *B*_*i*_ is unmodified. To authenticate a block ${\tilde{I}}_{i}=({\tilde{a}}_{i},{\tilde{b}}_{i},{\tilde{B}}_{i})$, the *w*-bit parity value

$${\tilde{p}}_{i}=({\tilde{b}}_{i}-{\tilde{a}}_{i})\mathrm{mod}2^{w}$$ (2)

is calculated, and the authentication code $\tilde{a}c_{i}^{w}=ac_{i}^{w}$ is regenerated. If ${\tilde{p}}_{i}\neq \tilde{a}c_{i}^{w}$, ${\tilde{I}}_{i}$ is judged as a tampered block. Otherwise, ${\tilde{I}}_{i}$ is judged as an untampered one. The pixels of the tampered block ${\tilde{I}}_{i}$ can be roughly recovered from the bitmaps of other untampered smooth blocks using the majority voting strategy. The detection and recovery procedures in detail can refer to [25].

## The proposed method

Hu et al.’s method uses the quantization levels and bitmaps of smooth blocks to embed authentication codes and recovery codes, respectively. Their method successfully detects and approximately recovers the tampered blocks, and the detection mainly relies on Eq (2). However, for any integer *ρ*, substituting ${\tilde{a}}_{i}\pm \rho \times 2^{w}$ or ${\tilde{b}}_{i}\pm \rho \times 2^{w}$ into Eq (2) also obtains the same ${\tilde{p}}_{i}$. Therefore, their method fails to detect the tampering by adding or subtracting *ρ*×2^*w*^ to the marked quantization levels. Moreover, the tampering of marked bitmaps also cannot be detected by their method because the modification of bitmaps does not alter the extracted authentication codes. Besides, the embedment of the *w*-bit authentication codes $ac_{i}^{w}$ into the quantization levels may cause a significant distortion for a large *w*. Therefore, the overflow and underflow problems can possibly occur. However, Hu et al.’s method provides no mechanisms to deal with these problems. Moreover, all pixels of a tampered block are recovered by a single mean value. As a result, the unpleasant mosaic-like effect is apparent, particularly in image edges.

**Fig 1:**
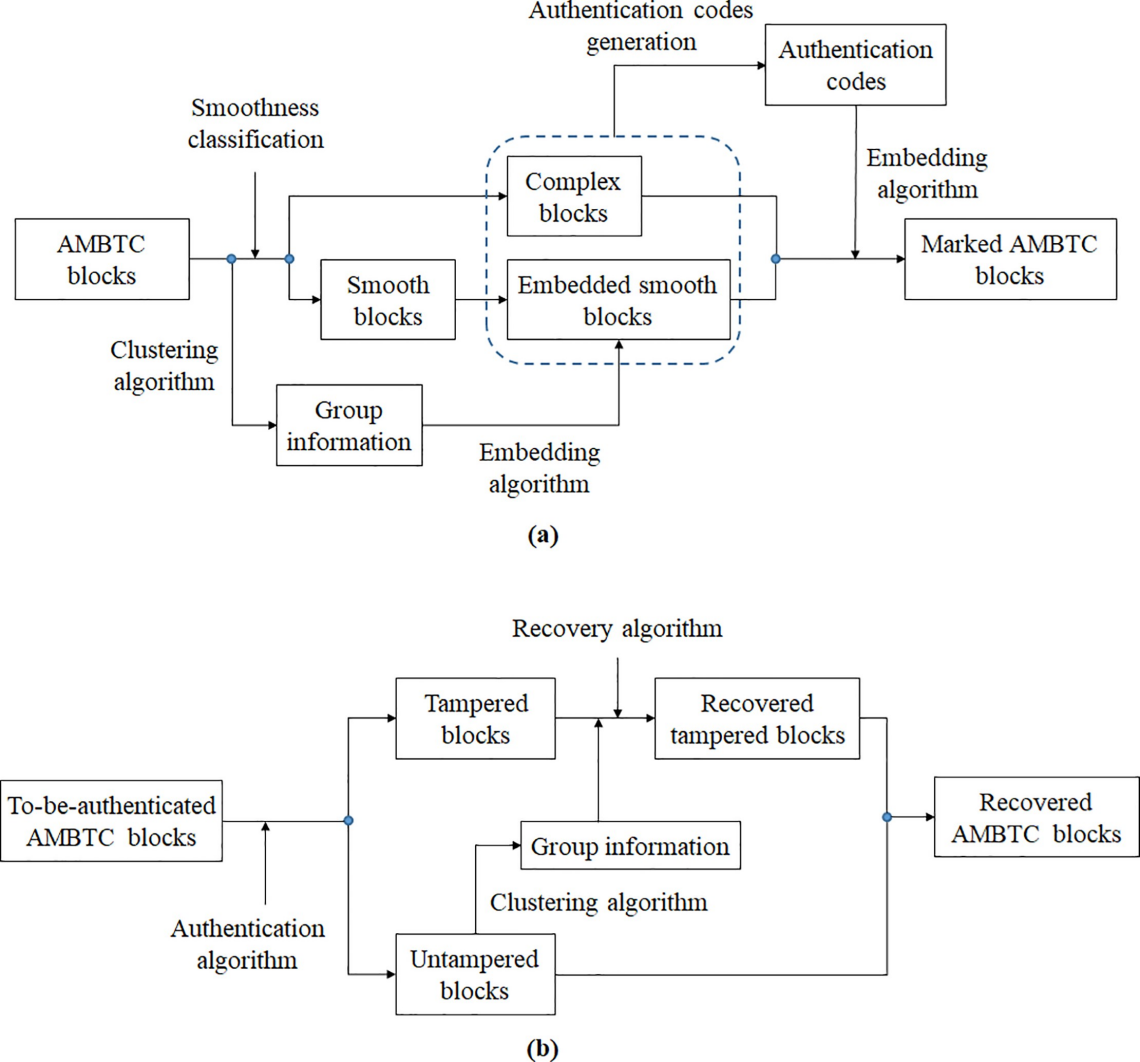
The framework of the proposed method. (a) The embedding procedures. (b) The authentication and recovery procedures.

We propose an improved authentication method with recovery capability for AMBTC codes to overcome the aforementioned problems. The framework of the proposed method is shown in Fig 1.

The proposed method divides the quantization levels into tuneable and embeddable portions. The embeddable portions of the quantization levels are used to carry the authentication codes generated from the features of blocks using a hash function. The bitmaps of the smooth blocks are employed to embed the recovery codes. The tuneable portions of quantization levels are fine-tuned to generate a set of authentication code candidates of length *w*. The best candidate that minimizes the distortion is embedded into embeddable portions of the quantization levels. To generate the recovery codes, the image blocks are classified into 2^*ψ*^ groups according to their similarities. The *τ* copies of group information are scrambled and embedded into bitmaps of smooth blocks. By comparing the authentication codes embedded in the quantization levels and the ones generated from the hash function, the tampered blocks can be detected. The tampered blocks can be recovered by averaging the untampered blocks of the same group with the aid of recovery codes. While comparing with prior works, the proposed method can detect all kinds of tamper and obtain a higher marked image quality. Most importantly, the proposed method has the capability to recover the tampered regions, and offers a very satisfactory recovered image quality. The authentication and recovery procedures in detail will be presented in the following sections.

### Generation and embedment of recovery codes

The recovery codes used in the proposed method are the *ψ*-bit indices generated using the k-means clustering algorithm [28], which gathers similar input vectors into one group by specifying an index ranging from 0 through 2^*ψ*^−1 to the group. Let ${\left\{I_{i}\right\}}_{i=1}^{N}$ be the image blocks decoded from the AMBTC codes ${\left\{(a_{i},b_{i},B_{i})\right\}}_{i=1}^{N}$. The proposed method takes ${\left\{I_{i}\right\}}_{i=1}^{N}$ as the input vectors and generates a set of group indices ${\left\{\sigma_{i}\right\}}_{i=1}^{N}$ as the outputs according to the similar measurement, where *σ*_*i*_∈[0,2^*ψ*^−1] and *ψ* is a user-specified constant (2^*ψ*^≪N). The group index *σ*_*i*_ represents that block *I*_*i*_ is clustered in the *σ*_*i*_-th group. Note that blocks with the same group index indicate that they are more similar than other blocks with different ones. The generated group indices ${\left\{\sigma_{i}\right\}}_{i=1}^{N}$ are the recovery information to be embedded into the bitmaps of smooth blocks.

The bitmap *B*_*i*_ of the smooth block *I*_*i*_ consists of *n*×*n* bits. The first *τ*×*ψ* bits are utilized to carry *τ*×*ψ*-bit recovery codes, and the other *n*×*n*−*τ*×*ψ* bits remain unmodified. To embed the recovery codes, the set ${\left\{\sigma_{i}\right\}}_{i=1}^{N}$ is scrambled using a key to generate *τ* copies of scrambled indices ${\left\{\sigma_{i}^{\prime (k)}\right\}}_{i=1}^{N}$ for 1≤*k*≤*τ*. The scrambled indices ${\left\{\sigma_{i}^{\prime (k)}\right\}}_{i=1}^{N}$ are then converted into *ψ*-bit binary representations ${\left\{{\left(\sigma_{i}^{\prime (k)}\right)}_{2}\right\}}_{i=1}^{N}$. To embed the indices, the AMBTC codes ${\left\{(a_{i},b_{i},B_{i})\right\}}_{i=1}^{N}$ are sequentially visited. If *b*_*i*_−*a*_*i*_≤*T*, where *T* is a predefined threshold, *I*_*i*_ is a smooth block and the bitmap *B*_*i*_ is used to embed ${\left\{\sigma_{i}^{\prime (k)}\right\}}_{k=1}^{\tau }$. The embedment can be conducted by replacing the ((*k*−1)×*ψ*+1)-th bit through (*k*×*ψ*)-th of *B*_*i*_ by ${\left(\sigma_{i}^{\prime (k)}\right)}_{2}$ for 1≤*k*≤*τ*. If *b*_*i*_−*a*_*i*_>*T*, *B*_*i*_ remains unchanged. Each block is processed in the same manner, and the embedded bitmap ${\left\{{\hat{B}}_{i}\right\}}_{i=1}^{N}$ can be obtained.

**Fig 2:**
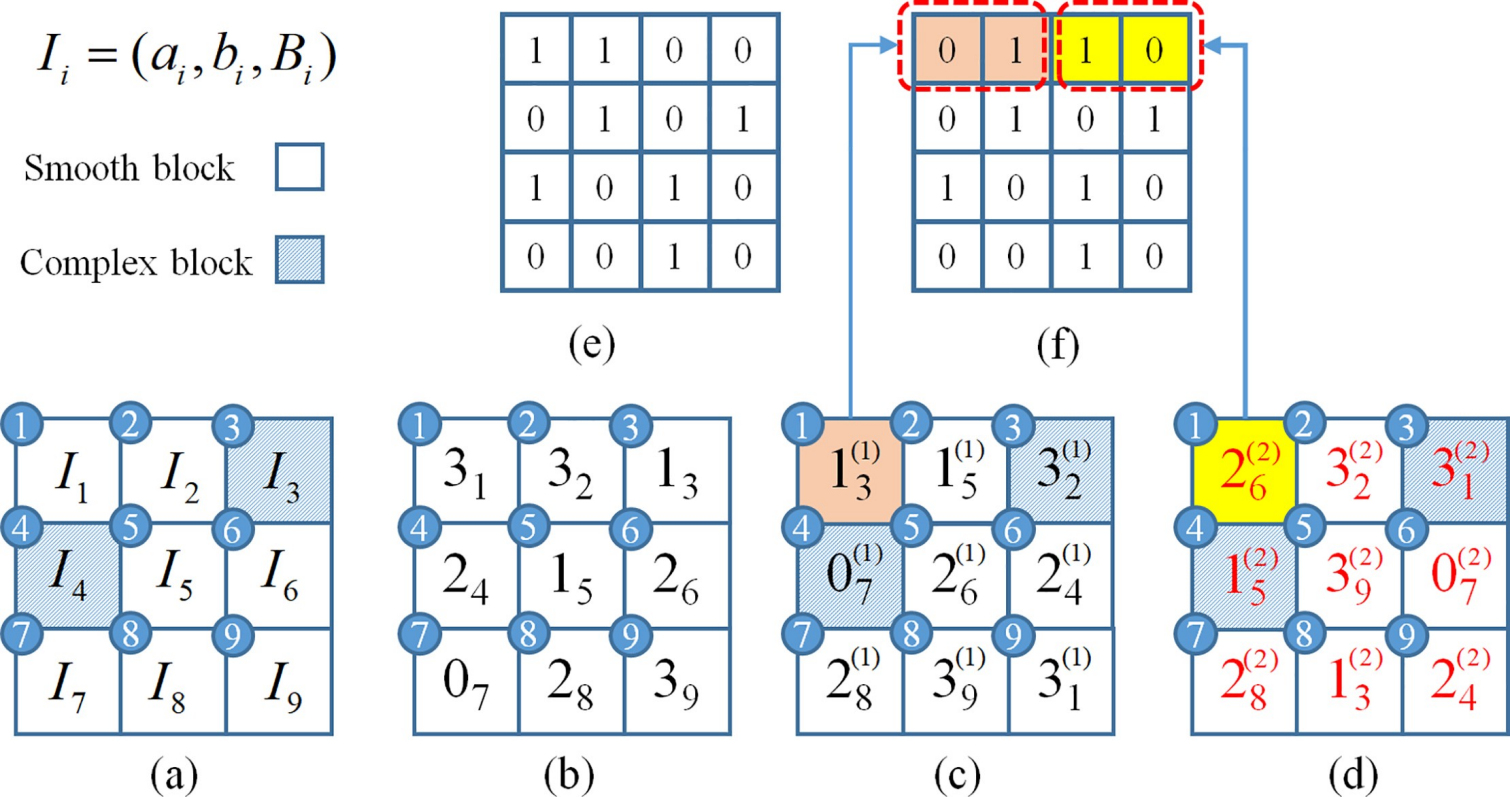
Illustration of the generation and embedment of recovery codes. (a) ${\left\{I_{i}\right\}}_{i=1}^{9}$. (b) ${\left\{\sigma_{i}\right\}}_{i=1}^{9}$. (c) ${\left\{\sigma_{i}^{\prime (1)}\right\}}_{i=1}^{9}$. (d) ${\left\{\sigma_{i}^{\prime (2)}\right\}}_{i=1}^{9}$. (e) *B*_1_. (f) ${\hat{B}}_{1}$.

We use a brief example to illustrate the generation and embedment procedures of the recovery codes. Let $I={\left\{I_{i}\right\}}_{i=1}^{9}$ be a 3×3 AMBTC compressed blocks (Fig 2A), where the third and fourth blocks are complex ones. Suppose *ψ* = 2 and *τ* = 2. Therefore, the nine blocks ${\left\{I_{i}\right\}}_{i=1}^{9}$ have to be classified into 2^*ψ*^ = 4 groups. Let Fig 2B be the classification results ${\left\{\sigma_{i}\right\}}_{i=1}^{9}$, where the subscripts indicate the positions of group indices. Because *τ*= 2, two scrambled copies of ${\left\{\sigma_{i}\right\}}_{i=1}^{9}$ are generated, as shown in Fig 2C and 2D. To embed the group information into the bitmaps of *I*, ${\left\{\sigma_{i}^{\prime (1)}\right\}}_{i=1}^{9}$ and ${\left\{\sigma_{i}^{\prime (2)}\right\}}_{i=1}^{9}$ are sequentially visited. Suppose Fig 2E is the bitmap *B*_1_ of block *I*_1_. The binary representations of $\sigma_{1}^{\prime (1)}$ and $\sigma_{1}^{\prime (2)}$ are 01_2_ and 10_2_, respectively. Therefore, we replace the first and second bits of *B*_1_ by 01_2_, and replace the third and fourth bits of *B*_1_ by 10_2_, as shown in Fig 2F.

The same embedding procedure is applied to the rest blocks, and we finish the embedment of recovery codes. Note that *I*_3_ and *I*_4_ are complex blocks, so no group indices are embedded into their bitmaps.

### Generation and embedment of authentication codes

Given two integers *x* and *y*, *x* can be expressed as the summation of two integers ⌊*x*/2^*y*^⌋×2^*y*^ and *x*mod2^*y*^. The first integer ⌊*x*/2^*y*^⌋×2^*y*^ is the dominant portion of *x*. The second integer *x*mod2^*y*^ is the decimal value of *y*-LSBs of *x*. In the proposed method, the dominant portions of *a*_*i*_ and *b*_*i*_ are used to generate a *w*-bit authentication code $ac_{i}^{w}$. The generated $ac_{i}^{w}$ is embedded into the LSBs of *a*_*i*_ and *b*_*i*_. Because the bitmaps of smooth blocks and the LSBs of quantization levels are modified for data embedment, a slight adjustment of the dominant portions of quantization levels may lead to a smaller distortion. Therefore, the dominant portions are indeed adjustable, and the LSBs of the quantization levels are embeddable portions. However, the adjustment of dominant portions also alters the generated $ac_{i}^{w}$. Therefore, we propose a least distortion embedding (LDE) technique to find the best dominant portion such that the embedment of $ac_{i}^{w}$ causes the smallest distortion.

Let $\left(a_{i},b_{i},{\hat{B}}_{i}\right)$ be the AMBTC tuple to be embedded with *w*-bit authentication code $ac_{i}^{w}$, where ${\hat{B}}_{i}$ is the bitmap derived from Section 3.1. Suppose $ac_{i}^{w_{1}}$ and $ac_{i}^{w_{2}}$ (*w*_1_+*w*_2_ = *w*) are the authentication codes to be embedded into the LSBs of *a*_*i*_ and *b*_*i*_, respectively, and $ac_{i}^{w}=ac_{i}^{w_{1}}\|ac_{i}^{w_{2}}$, where || represents the bitstream concatenation operator. The $ac_{i}^{w}$ is generated by hashing the small alteration of dominant portions of *a*_*i*_ and *b*_*i*_, the bitmap ${\hat{B}}_{i}$, the location information *i*, and the image identification number *I*_*d*_. Since the bitmaps of smooth blocks are utilized to carry recovery bits, a smooth block should be still classified into a smooth one after embedment. We use the function *f*(*a*_*i*_,*b*_*i*_) to evaluate whether a block *I*_*i*_ with the code $\left(a_{i},b_{i},{\hat{B}}_{i}\right)$ is smooth. If |*a*_*i*_−*b*_*i*_|≤*T*, *f*(*a*_*i*_,*b*_*i*_) returns 1, indicating that *I*_*i*_ is a smooth block; otherwise, *f*(*a*_*i*_,*b*_*i*_) returns 0. As a result, the embedment of authentication codes can be formulated as an optimization problem below:

$$\mathrm{Minimize}:\;\sum_{j=1}^{n\times n}{\left(I_{i,j}-I_{i,j}^{\prime }\right)}^{2}$$ (3)

$$\mathrm{Subject}\;\mathrm{to}:\;a_{i}^{d}=\left(\left\lfloor a_{i}/2^{w_{1}}\right\rfloor +c_{1}\right)\times 2^{w_{1}};\;b_{i}^{d}=\left(\left\lfloor b_{i}/2^{w_{2}}\right\rfloor +c_{2}\right)\times 2^{w_{2}};$$ (4)

$$ac_{i}^{w}={\mathrm{hash}}_{w}(a_{i}^{d},b_{i}^{d},i,{\hat{B}}_{i},I_{d});$$ (5)

$$a_{i}^{\prime }=a_{i}^{d}+{\left(ac_{i}^{w_{1}}\right)}_{10};\;b_{i}^{\prime }=b_{i}^{d}+{\left(ac_{i}^{w_{2}}\right)}_{10};$$ (6)

$$f(a_{i}^{\prime },b_{i}^{\prime })=f(a_{i},b_{i});$$ (7)

$$0\leq a_{i}^{\prime }\leq 255;\;0\leq b_{i}^{\prime }\leq 255;$$ (8)

$$|c_{1}|\leq c;|c_{2}|\leq c;\;c_{1},c_{2}\in \mathrm{Integers},$$ (9)

where hash_*w*_(•) is a *w*-bit hash function, and $I_{i,j}^{\prime }$ is the *j*-th pixel value of the *i*-th block $I_{i}^{\prime }$. In solving the problem, the constant *c* is confined within a small range, and setting *c* = 5 is enough to give the optimal solutions under insignificant computation cost. In some rare cases, if the optimal solutions cannot be found, we may simply perturb *a*_*i*_ and *b*_*i*_ by one unit iteratively and resolve the optimization problem until an optimal solution is found. We denote by $\left({\hat{a}}_{i},{\hat{b}}_{i}\right)$ the solution to the Eqs (3)–(9). Each block is processed in the same manner, and the marked AMBTC codes ${\left\{({\hat{a}}_{i},{\hat{b}}_{i},{\hat{B}}_{i})\right\}}_{i=1}^{N}$ can then be obtained.

Here is an example to illustrate the generation and embedment of authentication codes. Let the original AMBTC tuple be *I*_*i*_ = (*a*_*i*_,*b*_*i*_,*B*_*i*_) = (79,88,[1000;0101;1110;0011]), and the parameters used for the embedment are *w*_1_ = *w*_2_ = 2, *c* = 1, and *T* = 12. Let $\left(a_{i},b_{i},{\hat{B}}_{i})=(79,88,[0101;0011;1010;1011]\right)$ be the tuple obtained by using the embedding technique described in Section 3.1. Because $\left\lfloor a_{i}/2^{w_{1}}\right\rfloor =\left\lfloor 79/2^{2}\right\rfloor =19$ and $\left\lfloor b_{i}/2^{w_{2}}\right\rfloor =\left\lfloor 88/2^{2}\right\rfloor =22$, we have nine pairs of $\left(a_{i}^{d},b_{i}^{d}\right)$ according to Eqs (4) and (9). These nine pairs are (72,84), (72,88), (72,92), (76,84), (76,88), (76,92), (80,84), (80,88), and (80,92). Suppose the authentication codes generated by using these pairs and other information are 0010_2_, 1010_2_, 1100_2_, 1011_2_, 1000_2_, 1101_2_, 1100_2_, 0101_2_, and 1001_2_. According to Eq (6), the nine pairs of $\left(a_{i}^{\prime },b_{i}^{\prime }\right)$ are (72,86), (74,90), (75,92), (78,87), (78,88), (79,93), (83,84), (81,89), and (82,93). Among these nine candidate pairs, five pairs (78,87), (78,88), (83,84), (81,89), and (82,93) satisfy the constraint given in Eq (7). By using these five candidate pairs and bitmap ${\hat{B}}_{i}$ to construct five AMBTC blocks ${\left\{I_{i}^{\prime (k)}\right\}}_{k=1}^{5}$, and calculating the square errors between these five blocks and the block *I*_*i*_ constructed from the tuple (*a*_*i*_,*b*_*i*_,*B*_*i*_), we have 565, 628, 319, 568 and 1053. Because the square error between $I_{i}^{\prime (3)}$ and *I*_*i*_ is the smallest, we have $\left(a_{i}^{\prime },b_{i}^{\prime })=(83,84\right)$. Therefore, the marked AMBTC tuple $\left({\hat{a}}_{i},{\hat{b}}_{i},{\hat{B}}_{i})=(83,84,[0101;0011;1010;1011]\right)$ can be obtained.

### Detection and recovery of the tampered blocks

To authenticate the AMBTC tuple ${\left\{({\tilde{a}}_{i},{\tilde{b}}_{i},{\tilde{B}}_{i})\right\}}_{i=1}^{N}$ with the image identification ${\tilde{I}}_{d}$, the embedded authentication code $\tilde{e}ac_{i}$ is extracted by concatenating the *w*_1_ LSBs of ${\tilde{a}}_{i}$ and *w*_2_ LSBs of ${\tilde{b}}_{i}$. The *w*-bit hash value of the tuple $\left({\tilde{a}}_{i},{\tilde{b}}_{i},{\tilde{B}}_{i}\right)$ can be calculated by $\tilde{a}c_{i}^{w}={\mathrm{hash}}_{w}(\left\lfloor {\tilde{a}}_{i}/2^{w_{1}}\right\rfloor \times 2^{w_{1}},\left\lfloor {\tilde{b}}_{i}/2^{w_{2}}\right\rfloor \times 2^{w_{2}},i,{\tilde{B}}_{i},{\tilde{I}}_{d})$. If $\tilde{e}ac_{i}=\tilde{a}c_{i}^{w}$, the tuple $\left({\tilde{a}}_{i},{\tilde{b}}_{i},{\tilde{B}}_{i}\right)$ is judged as an untampered one. Otherwise, $\left({\tilde{a}}_{i},{\tilde{b}}_{i},{\tilde{B}}_{i}\right)$ has been tampered. Each tuple in the AMBTC codes is authenticated in a similar manner, and the tampered tuples can be detected. The aforementioned detection procedures are referred to as the first-stage detection. The proposed method also uses the second-stage detection to further refine the detection results. Since most of the tampered regions are contiguous, a block judged as an untampered one is likely to be tampered if this block is surrounded by some tampered blocks. Therefore, after the first-stage detection, if two out of the four neighbors of an untampered block are tampered, this untampered block is re-judged as a tampered one.

After the second-stage detection, the location information of the tampered and untampered blocks is known. Since *τ* copies of the group information (indices) of those tampered blocks are scrambled and embedded in the bitmaps of smooth blocks, we can extract *τ* copies of the recovery information ${\left\{{\tilde{\sigma }}_{i}^{\prime (k)}\right\}}_{i=1}^{N}$, and perform the reverse scrambling using the same key to obtain the group information ${\left\{{\tilde{\sigma }}_{i}^{\left(k\right)}\right\}}_{i=1}^{N}$ for 1≤*k*≤*τ*. Note that some of the indices in ${\left\{{\tilde{\sigma }}_{i}^{\left(k\right)}\right\}}_{i=1}^{N}$ might be damaged or missing because they are mapped from the tampered smooth blocks or complex ones. By comparing *τ* copies of ${\left\{{\tilde{\sigma }}_{i}^{\left(k\right)}\right\}}_{k=1}^{\tau }$, the correct indices ${\left\{{\tilde{\sigma }}_{i}\right\}}_{i=1}^{N}$ associated with blocks ${\left\{{\tilde{I}}_{i}\right\}}_{i=1}^{N}$ can be obtained, though some of them might be still damaged or missing. We use ${\left\{t_{i}\right\}}_{i=1}^{N}$ to represent the detection results, where *t*_*i*_ = 0 if ${\tilde{I}}_{i}$ is tampered, and *t*_*i*_ = 1 if ${\tilde{I}}_{i}$ is untampered. The recovered block ${\tilde{I}}_{i}^{R}$ used to replace the corresponding tampered block ${\tilde{I}}_{i}^{T}$ can be obtained by averaging those untampered ones with the same group index ${\tilde{\sigma }}_{i}^{}$. That is, suppose there are $N_{{\tilde{\sigma }}_{i}}^{UT}$ untampered blocks with group index valued ${\tilde{\sigma }}_{i}$, then the tampered block ${\tilde{I}}_{i}^{T}$ can be recovered by

$${\tilde{I}}_{i}^{R}=\frac{1}{N_{{\tilde{\sigma }}_{i}}^{UT}}\underset{t_{j}=1,{\tilde{\sigma }}_{j}={\tilde{\sigma }}_{i}}{\sum_{j=1}^{N}{\tilde{I}}_{j}}.$$ (10)

The aforementioned recovery procedures are referred to as the first-stage recovery of the proposed method. Notice that some group indices in ${\left\{{\tilde{\sigma }}_{i}\right\}}_{i=1}^{N}$ might not be available. Therefore, tampered blocks with those unavailable group indices cannot be recovered. Fortunately, these blocks are sparsely distributed in the tampered regions. For those tampered blocks, the second-stage recovery can be performed by averaging the block means of their available neighboring blocks.

**Fig 3:**
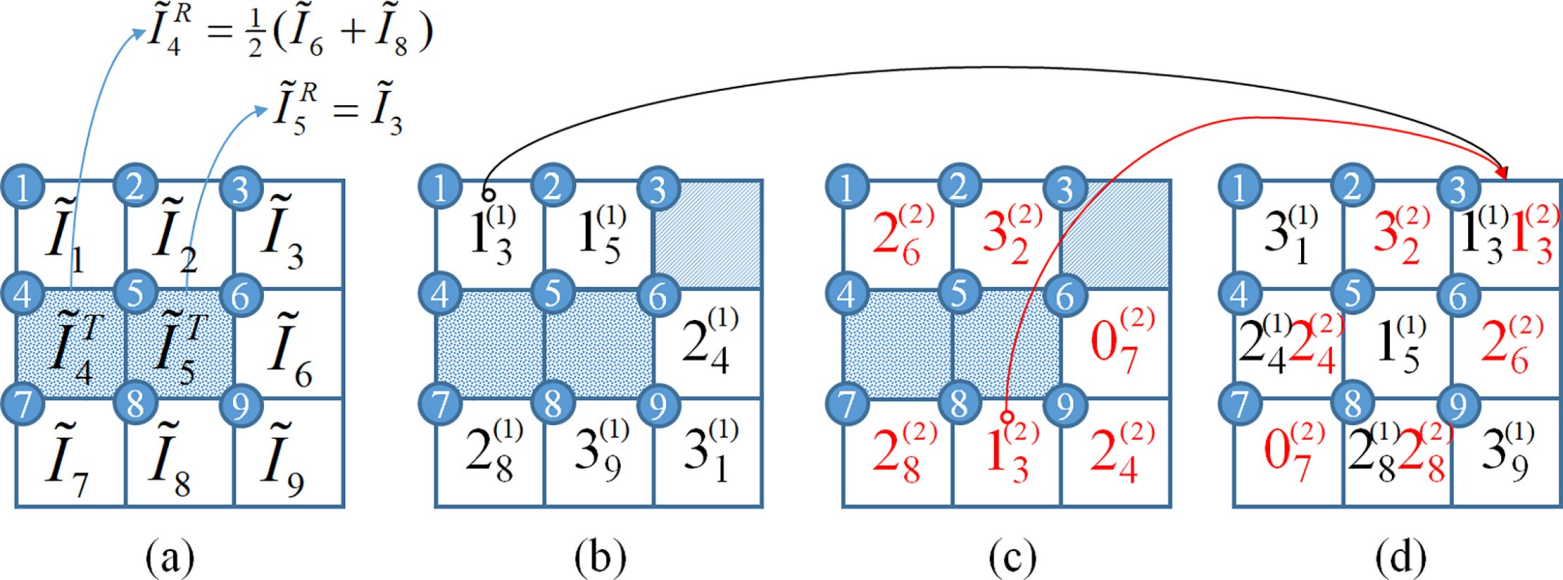
Illustration of the recovery of tampered blocks.

We continue the example given in Section 3.1 to illustrate the recovery of tampered blocks. Suppose the fourth and fifth blocks have been detected as tampered ones (Fig 3A. Note that the third and fourth blocks are complex ones; therefore, the bitmaps of these blocks have no recovery codes embedded. By extracting the recovery codes embedded in the bitmaps of other blocks, we obtain two copies of the scrambled group indices (Fig 3B and 3C). Perform the inverse scrambling of these indices, we have the recovered group indices ${\left\{{\tilde{\sigma }}_{i}\right\}}_{i=1}^{9}$ (Fig 3D). Because the group index of ${\tilde{I}}_{4}$ is ${\tilde{\sigma }}_{4}=2$, and the indices of the sixth and eighth blocks are also equal to 2, ${\tilde{I}}_{4}$ can be recovered by averaging ${\tilde{I}}_{6}$ and ${\tilde{I}}_{8}$. Similarly, because the group index of ${\tilde{I}}_{5}$ is ${\tilde{\sigma }}_{5}=1$ and only the index of the third block is equal to 1, ${\tilde{I}}_{5}$ is then recovered by ${\tilde{I}}_{3}$.

## Experimental results

**Fig 4:**
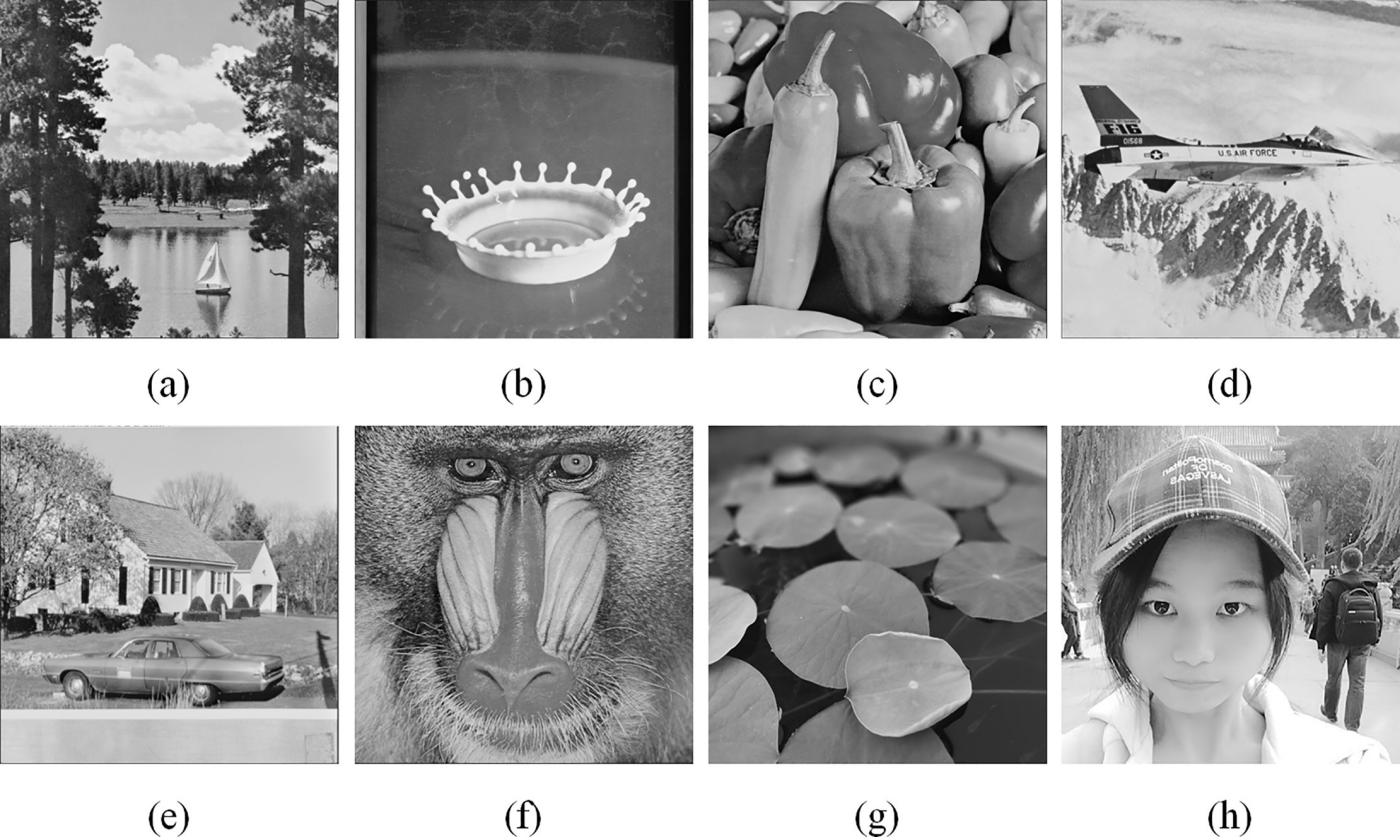
Eight test images. (a) Sailboat. (b) Splash. (c) Peppers. (d) Jet. (e) House. (f) Baboon. (g) Leaves. (h) Girl.

In this section, we conduct several experiments to evaluate the performance of the proposed method and compare the results with some related works. A total of eight grayscale images of size 512×512 are used as the test images (Fig 4). In these images, the first six images are obtained from the USC-SIPI image database [29], while the last two images are taken from a modern digital camera. These test images are compressed using the AMBTC compression technique with a 4×4 block size. The compressed codes are then used to embed the authentication and recovery codes.

The peak signal-to-noise ratio (PSNR) metric is used to measure the quality of the marked image. A higher PSNR indicates that the image has a better visual quality. The PSNR presented in this section is obtained by comparing the marked image or recovered image with the image decoded from the original unmodified AMBTC codes.

Since the original AMBTC codes ${\left\{a_{i},b_{i},B_{i}\right\}}_{i=1}^{N}$ are modified due to the embedment of the authentication and recovery codes, the distortions can be theoretically analyzed by comparing ${\left\{a_{i},b_{i},B_{i}\right\}}_{i=1}^{N}$ and the marked codes ${\left\{{\hat{a}}_{i},{\hat{b}}_{i},{\hat{B}}_{i}\right\}}_{i=1}^{N}$ for a given threshold *T*. Let $\eta_{i}^{uv}$ be the number of bits valued *u* in *B*_*i*_ but valued *v* in ${\hat{B}}_{i}$ after embedment. Therefore, the mean square error (MSE) can be obtained by

$$\begin{array}{l} MSE=\frac{1}{N\times n^{2}}(\sum_{\begin{array}{c} i=1\\ b_{i}-a_{i}\leq T \end{array}}^{N}\left(\eta_{i}^{00}{\left(a_{i}-{\hat{a}}_{i}\right)}^{2}+\eta_{i}^{11}{\left(b_{i}-{\hat{b}}_{i}\right)}^{2}+\eta_{i}^{01}{\left(a_{i}-{\hat{b}}_{i}\right)}^{2}+\eta_{i}^{10}{\left(b_{i}-{\hat{a}}_{i}\right)}^{2}\right)\\ \;+\sum_{\begin{array}{c} i=1\\ b_{i}-a_{i}>T \end{array}}^{N}\left(\eta_{i}^{00}{\left(a_{i}-{\hat{a}}_{i}\right)}^{2}+\eta_{i}^{11}{\left(b_{i}-{\hat{b}}_{i}\right)}^{2}\right))\\ =\frac{1}{N\times n^{2}}(\sum_{i=1}^{N}\left(\eta_{i}^{00}{\left(a_{i}-{\hat{a}}_{i}\right)}^{2}+\eta_{i}^{11}{\left(b_{i}-{\hat{b}}_{i}\right)}^{2}\right)+\sum_{\begin{array}{c} i=1\\ b_{i}-a_{i}\leq T \end{array}}^{N}\left(\eta_{i}^{01}{\left(a_{i}-{\hat{b}}_{i}\right)}^{2}+\eta_{i}^{10}{\left(b_{i}-{\hat{a}}_{i}\right)}^{2}\right)). \end{array}$$ (11)

As seen from Eq (11), a smaller *T* can obtain a higher quality of the marked image. However, the quality of the recovered image could be reduced due to insufficient embedment of recovery codes. It is interesting to note that for a given threshold *T*, the smooth images possess larger distortions because the blocks with *b*_*i*_−*a*_*i*_≤*T* used to carry the recovery codes are more than those of complex ones.

### Performance comparisons of the proposed method

Table 2 shows the image quality comparisons of the eight test images when setting *w*_1_ = *w*_2_ = 2 and *T* = 12 for various combinations of *ψ* and *τ*. Note that the bitmap of a 4×4 block consists of 16 bits, and *τ* copies of *ψ*-bit recovery codes are chosen such that most of the bits in the bitmap of smooth blocks are embedded (*τ*×*ψ*≤16). As shown in Table 2, all the marked images achieve satisfactory image quality. Note that setting *τ* = 2 and *ψ* = 6 give the highest image quality for all the test images, since the number of bits modified in the bitmap is the smallest. It is interesting to note that under the same threshold *T* = 12, the PSNRs of complex images such as Baboon are higher than those smooth ones (e.g., the Splash image). This is because the smooth blocks of a complex image are fewer than those of a smooth image. The proposed method uses bitmaps of smooth blocks to carry the recovery information, and thus the distortion of complex images is smaller because fewer recovery codes are carried.

**Table 2:** PSNR comparisons of fully embedded images.

*τ*	*ψ*	Sailboat	Splash	Peppers	Jet	House	Baboon	Leaves	Girl
**5**	**3**	38.79	39.15	38.09	40.53	40.74	40.54	40.72	40.37
**4**	**4**	38.80	39.19	38.07	40.49	40.74	40.55	40.69	40.39
**3**	**5**	38.78	39.21	38.09	40.49	40.73	40.53	40.70	40.41
**2**	**6**	38.96	39.38	38.27	40.68	40.89	40.69	40.84	40.59
**2**	**7**	38.80	39.22	38.11	40.52	40.77	40.56	40.72	40.44
**2**	**8**	38.78	39.19	38.08	40.50	40.72	40.54	40.71	40.41

Table 3 shows the effect of the LDE technique on image quality by varying the length of authentication codes *w*. In this experiment, *ψ* = 7, *τ* = 2 and *T* = 12 are set. The LDE technique effectively increases the image quality for every *w* and every test image. It is worth noting that the increase in PSNRs is more substantial at smaller *w*, and the increase becomes smaller as *w* increases. For example, the increase in PSNR of the Sailboat image at *w* = 2 is 39.50−36.87 = 2.63dB, while the increase reduces to 32.38−31.47 = 0.91dB when *w* = 8. The reason is that the embedment using a larger *w* distorts the quantization levels more, and a smooth block is likely to become a complex one or vice versa after embedment. Therefore, the constraint given in Eq (7) has to be applied to maintain the consistency of smoothness classification. As a result, the increase in PSNR is penalized as the length of the authentication codes increases.

**Table 3:** PSNR comparisons with and without the LDE technique.

*w*	Method	Sailboat	Splash	Peppers	Jet	House	Baboon	Leaves	Girl
**2**	**w/o LDE**	36.87	37.25	36.04	38.94	39.36	39.19	39.22	38.97
	**with LDE**	39.50	39.99	38.71	41.61	41.93	41.69	41.89	41.54
**4**	**w/o LDE**	36.58	36.76	35.78	38.19	38.61	38.53	38.39	38.27
	**with LDE**	38.80	39.22	38.11	40.52	40.77	40.56	40.72	40.44
**6**	**w/o LDE**	35.08	35.22	34.61	36.04	36.09	36.09	36.12	35.93
	**with LDE**	36.64	37.02	36.26	37.73	37.73	37.61	37.85	37.56
**8**	**w/o LDE**	31.47	31.83	31.58	31.75	31.70	31.43	31.89	31.46
	**with LDE**	32.38	32.62	32.29	32.79	32.83	32.65	32.96	32.66

Table 4 shows the PSNR comparisons of a variety of threshold *T* at *ψ* = 7, *τ* = 2, and *w* = 4. It is not surprising that the PSNR decreases as *T* increases, owing to an increase of *T* which causes more blocks to be classified into smooth ones. Since more smooth blocks are allowed to embed more recovery codes into the bitmaps, the degradation in image quality can be observed. Nevertheless, all the marked images still have a satisfactory image quality, even when *T* = 20 is set.

**Table 4:** PSNR comparisons of a variety of thresholds.

*T*	Sailboat	Splash	Peppers	Jet	House	Baboon	Leaves	Girl
**4**	44.80	43.44	44.33	43.98	44.73	45.58	43.89	44.83
**8**	41.66	40.50	40.25	42.13	42.86	43.77	41.95	42.85
**12**	38.80	39.22	38.11	40.52	40.77	40.56	40.72	40.44
**16**	37.21	38.31	36.94	39.05	38.61	38.09	39.73	38.39
**20**	35.90	37.73	36.03	37.74	36.74	36.28	38.92	36.63

**Fig 5:**
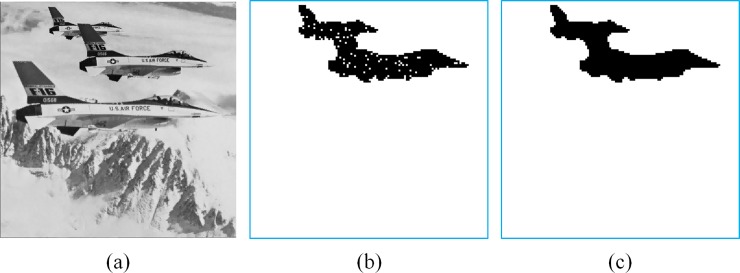
Detection of a tampered image. (a) Tampered Jet image. (b) First stage detection. (c) Second stage detection.

To demonstrate the detectability of the proposed method, we tamper the Jet image by adding two additional jets (Fig 5A). In the experiment, the parameters *T* = 12, *ψ* = 7, *τ* = 2, and *w* = 4 are set. The first-stage detection result is shown in Fig 5B, where the tampered blocks are marked in black. Because *w* = 4, the theoretical probability of hash collision is 1/2^4^ = 0.0625. In this experiment, 1343 blocks are tampered, and 1260 blocks are detected. Therefore, the false negative rate is approximately 1−1260/1343≃0.0618, which coincides with the theoretical value. The second-stage detection is able to detect all the tampered blocks (Fig 5C).

**Fig 6:**
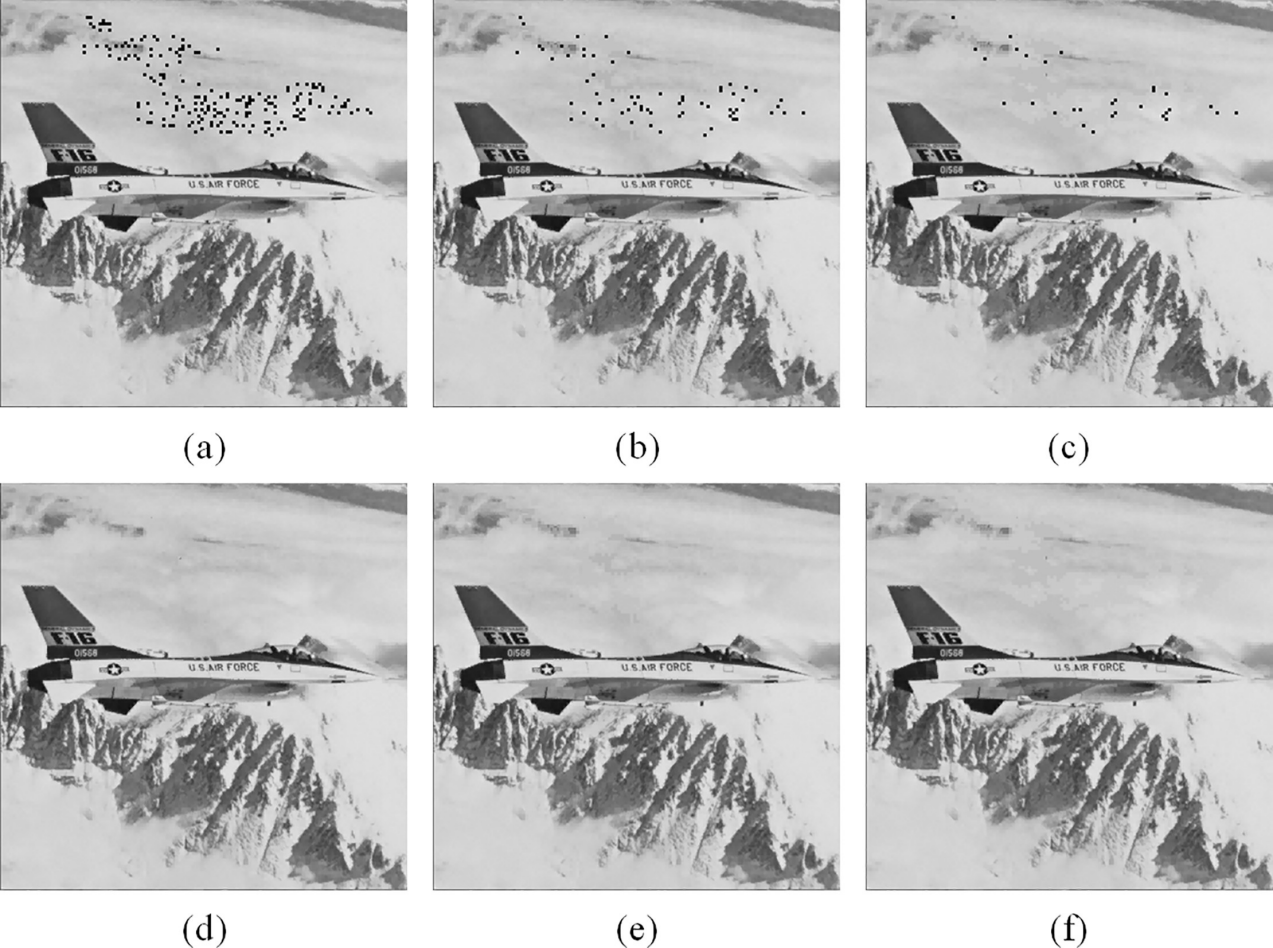
Comparisons of the recovery results. (a) First-stage recovery *τ* = 2,*ψ*= 7. (b) First-stage recovery *τ* = 3,*ψ* = 5. (c) First-stage recovery *τ* = 4,*ψ* = 4. (d) Second-stage recovery *τ* = 2,*ψ* = 7. (e) Second-stage recovery *τ* = 3,*ψ* = 5. (f) Second-stage recovery *τ* = 4,*ψ* = 4.

Fig 6 demonstrates the recovery capability of the proposed method when embedding two, three and four copies of recovery codes. Fig 6A–6C give the results of the first-stage recovery, where the black dots represent unrecoverable tampered blocks. Note that some blocks appointed to embed the recovery codes of tampered blocks are complex or tampered. Therefore, the recovery codes of these blocks are unavailable. As a result, the presence of unrecoverable tampered blocks is likely inevitable.

Not surprisingly, the unrecoverable blocks decrease as *τ* increases. Since more copies of the recovery codes are embedded into the bitmaps of smooth blocks, a tampered block has more chances to be recovered by the undamaged codes. However, more copies of recovery codes are also accompanied by a shorter length of recovery codes due to the limited bitmap size, and subsequently impede the quality of recovered portions. As shown in Fig 6D–6F where the second-stage detection results are presented, the visual quality of Fig 6D is noticeably better than those of Fig 6E and 6F. Notice that the contour effect is apparent in the recovered portion of clouds in Fig 6F; however, it is unnoticeable in Fig 6D. In fact, the PSNRs of the recovered Jet images shown in Fig 6D, 6E and 6F are 40.32, 40.08 and 39.84 dB, respectively, concurring with the visual perception. In the proposed scheme, we recommend setting *τ* = 2 to achieve a more satisfactory recovery result.

### Comparisons with Hu et al.’s method

In this section, we compare the marked image quality, detectability, and recovered image quality of the proposed method with those of Hu et al.’s method. To make a fair comparison, we set *T* = 12, *τ* = 2, and *ψ* = 8 for both methods. Table 5 shows the image quality comparisons for different lengths of authentication codes.

**Table 5:** Image quality comparisons with Hu et al.’s method.

*w*	Image	Sailboat	Splash	Peppers	Jet	House	Baboon	Leaves	Girl
**2**	Proposed	39.43	39.93	38.64	41.53	41.84	41.62	41.85	41.49
	Hu et al.	36.30	36.72	35.44	38.39	38.88	38.63	38.67	38.36
**3**	Proposed	39.23	39.67	38.46	41.17	41.44	41.26	41.39	41.07
	Hu et al.	36.26	36.41	35.33	38.10	38.52	38.46	38.34	38.16
**4**	Proposed	38.78	39.19	38.08	40.50	40.72	40.54	40.71	40.41
	Hu et al.	36.00	36.15	35.41	37.16	37.40	37.41	37.36	37.25
**5**	Proposed	37.91	38.25	37.31	39.27	39.38	39.27	39.43	39.16
	Hu et al.	32.44	32.86	32.37	33.07	32.89	32.92	33.25	32.86
**6**	Proposed	36.64	37.00	36.26	37.69	37.69	37.58	37.83	37.57
	Hu et al.	25.45	25.40	25.29	25.56	25.60	26.07	25.42	25.45

As shown in Table 5, the image qualities of the proposed method are all higher than those of Hu et al.’s method, and the improvement in PSNR is even significant for a larger *w*. For example, when *w* = 2, the improvement in PSNR for the Sailboat image is 39.43−36.30 = 3.13 dB, whereas the improvement increases 36.64−25.45 = 11.19 dB when *w* = 16 is set. The rapid decrease in PSNR of Hu et al.’s method is due to the fact that the parity value *p*_*i*_ given in Eq (1) has to satisfy $p_{i}=ac_{i}^{w}$, where $ac_{i}^{w}$ is the to-be-embedded authentication code. As a result, a substantial modification to the quantization levels is inevitable. Moreover, Hu et al.’s method provides no mechanism to solve the overflow and underflow problems. Therefore, once the embedded quantization levels are overflow or underflow, the embedded messages cannot be extracted. In contrast, the proposed method has provided a constraint (Eq (8)) to prevent these problems from occurring.

**Fig 7:**
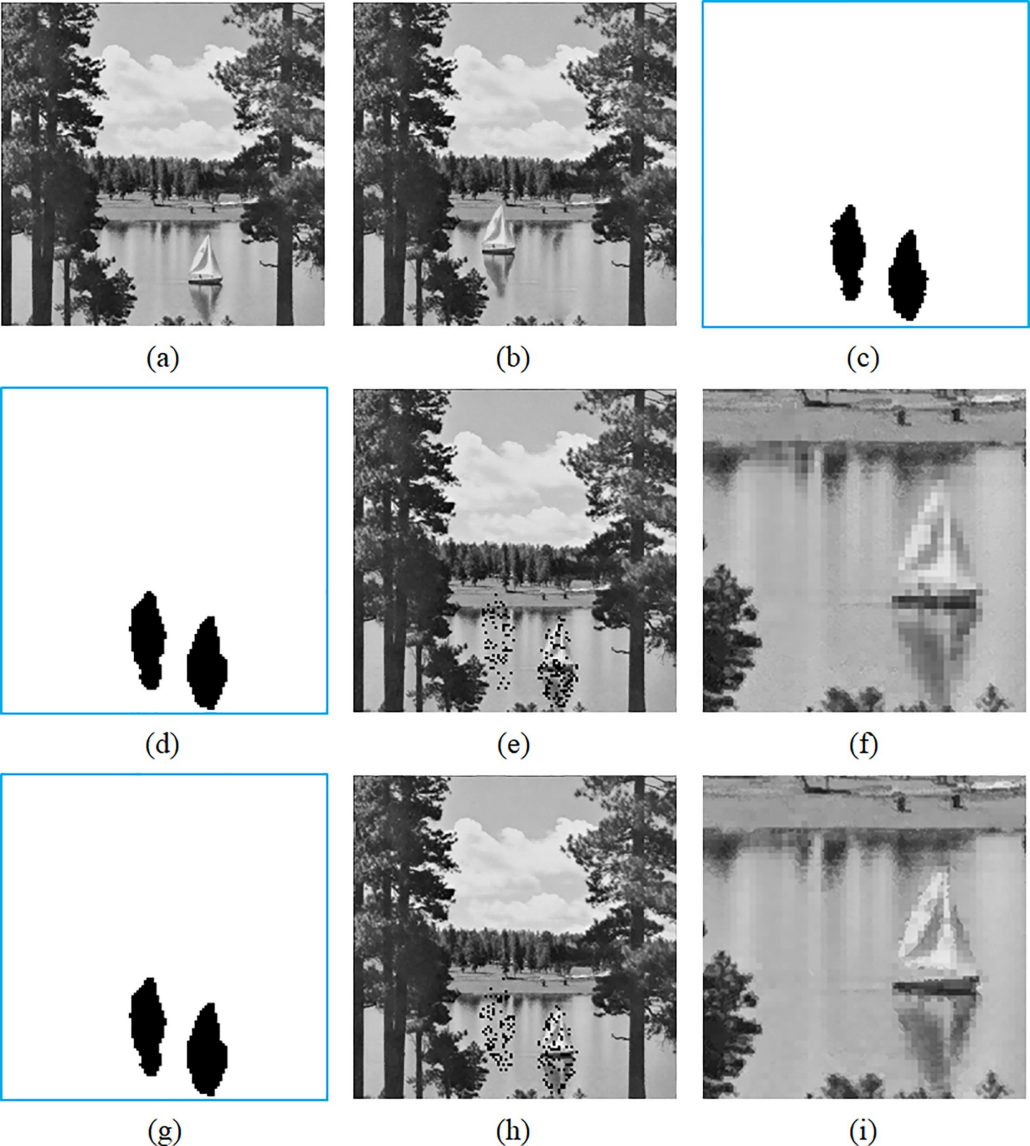
Comparisons of the detection and recovery results. (a) Marked image. (b) Tampered image. (c) Tampered regions. (d) Second-stage detection of Hu et al.’s method. (e) First-stage recovery of Hu et al.’s method. (f) Second-stage recovery of Hu et al.’s method. (g) Second-stage detection of the proposed method. (h) First-stage recovery of the proposed method. (i) Second-stage recovery of the proposed method.

In the following experiments, we compare the detectability and recoverability of both methods. The length of authentication code *w* = 4 is set for both methods to obtain a fair comparison between the image quality and detectability. To tamper the marked Sailboat image (Fig 7A), we move the boat on the river to the northwest of its original location. The background of the boat is also modified to make the scenes look more natural. The tampered image is shown in Fig 7B, while tampered regions are indicated in Fig 7C. Both of Hu et al.’s and the proposed methods are able to detect the tampering of the boat (Fig 7D and 7G), and recover most of the tampered blocks (Fig 7E and 7H). However, the recovered blocks of Hu et al.’s method have apparent mosaic-like patterns, as shown in Fig 7F, which is the magnified version of the Sailboat image. This is because all pixels of a tampered block are recovered by an identical mean value. In contrast, the tampered blocks of the proposed method are recovered by averaging the similar blocks of tampered ones. As a result, a more pleasant recovery result can be achieved (Fig 7I). In fact, the PSNRs of the images shown in Fig 7F and 7I are 34.42 dB and 37.35 dB, respectively, revealing that the image quality of the proposed method is better than that of Hu et al.’s method.

### Performance comparisons of some special tampering

**Fig 8:**
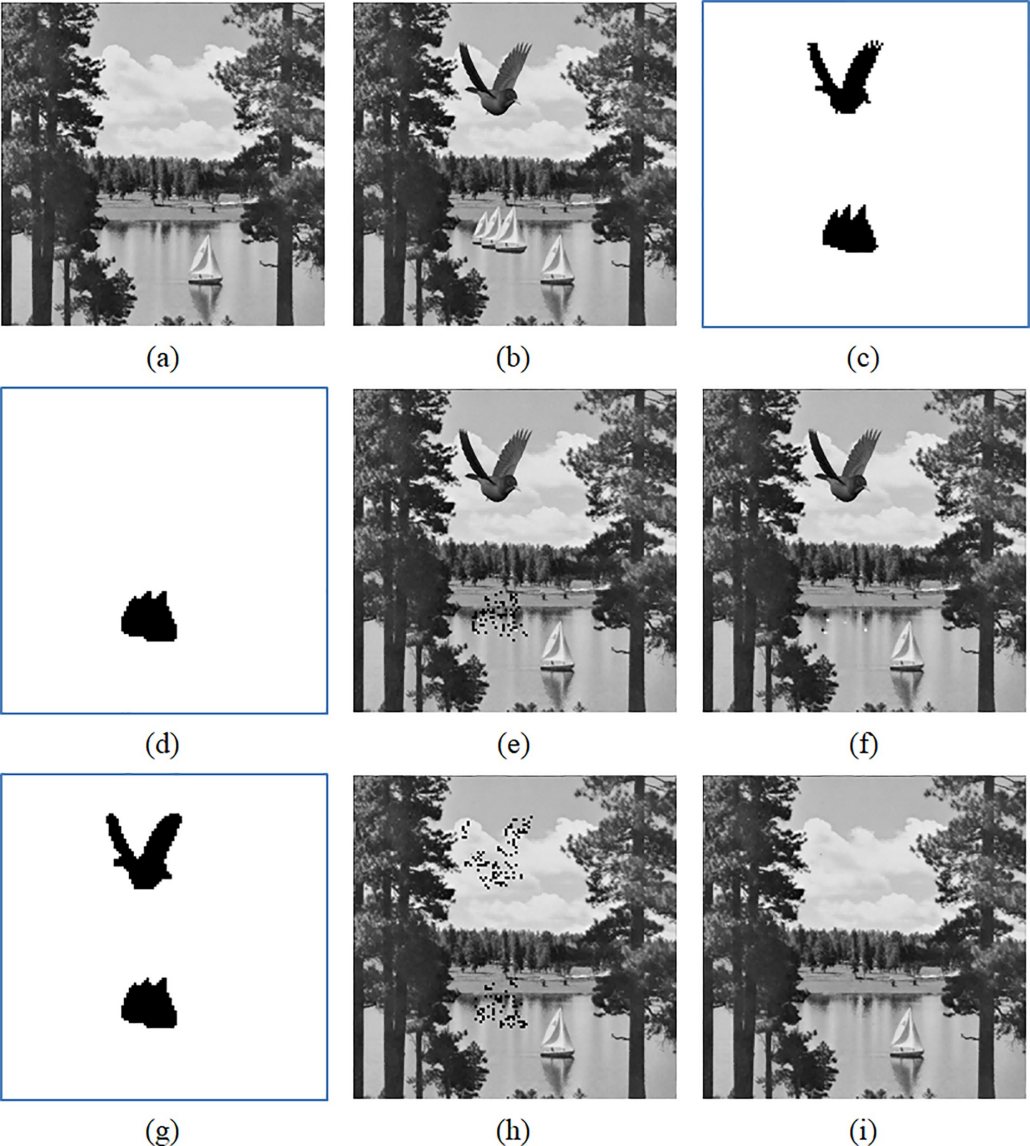
Comparisons of the detection and recovery results for a special tampering. (a) Marked image. (b) Tampered image. (c) Tampered regions. (d) Second-stage detection of Hu et al.’s method. (e) First-stage recovery of Hu et al.’s method. (f) Second-stage recovery of Hu et al.’s method. (g) Second-stage detection of the proposed method. (h) First-stage recovery of the proposed method. (i) Second-stage recovery of the proposed method.

A well-designed authentication scheme should be able to detect any kinds of tampering to a large extent. In this section, we perform special tampering to the marked Sailboat image obtained from Hu et al.’s and the proposed methods. In the experiment, the parameters *T* = 12, *ψ* = 8, *τ* = 2, and *w* = 4 are set for both methods. To tamper the marked image shown in Fig 8A, we paste a bird image in the sky and paste three boats on the river. The tampered image and tampered regions are shown in Fig 8B and 8C, respectively. The tuples ${\left\{({\hat{a}}_{i}^{\prime },{\hat{b}}_{i}^{\prime },{\hat{B}}_{i}^{\prime })\right\}}_{i=1}^{N_{bird}}$ of the pasted bird image are specially designed to replace the tuples ${\left\{({\hat{a}}_{i},{\hat{b}}_{i},{\hat{B}}_{i})\right\}}_{i=1}^{N_{bird}}$ at the same position of the marked Sailboat image, where *N*_*bird*_ is the total number of blocks of the bird image. To generate ${\hat{I}}_{i}^{\prime }=({\hat{a}}_{i}^{\prime },{\hat{b}}_{i}^{\prime },{\hat{B}}_{i}^{\prime })$, let $I_{i}^{bird}=(\alpha_{i},\beta_{i},\Gamma_{i})$ be a tuple of the original bird image and set ${\hat{B}}_{i}^{\prime }=\Gamma_{i}$. In addition, the quantization levels ${\hat{a}}_{i}^{\prime }$ and ${\hat{b}}_{i}^{\prime }$ of the pasted bird image should satisfy $({\hat{b}}_{i}^{\prime }-{\hat{a}}_{i}^{\prime })\mathrm{mod}2^{w}=({\hat{b}}_{i}-{\hat{a}}_{i})\mathrm{mod}2^{w}$, and the squared error between $I_{i}^{bird}$ and ${\hat{I}}_{i}^{\prime }$ is the smallest.

Fig 8D gives the detection result using Hu et al.’s method, indicating that their method fails to detect the tampering of the bird image. Fig 8E and 8F give the recovery results of the first and second stages. As shown in Fig 8F, the bird image cannot be recovered, while some incorrect recovered blocks are sparsely distributed in the tampered regions of boats. The incorrect recovery is due to the misjudgment of the tampered blocks of the bird image, and the recovery codes of the regions tampered by the boats image is partially embedded in the bitmaps of the bird image. In contrast, the proposed method not only successfully detects both of the tampered regions (Fig 8G) but also gives a satisfactory recovery result (Fig 8H and 8I).

Table 6 shows the performance comparisons of the proposed and relevant works. In this table, the term “Type A tampering” indicates that the tampering is performed by flipping two bits of the bitmap, whereas “Type B tampering” represents the tampering is conducted by adding a constant 16 to the quantization levels. Note that only [22] and the proposed method are able to detect the aforementioned special tampering, while the other methods are not. The reason is that methods [19–21] and [23–25] use a PRNG to generate the authentication codes, which are irrelevant to the contents they protect. In contrast, the authentication codes of [22] and the proposed method are generated by hashing the to-be-protected contents. Therefore, they are able to detect various types of tampering. Note that of all the compared methods, only [25] and the proposed method offer the capability to recover the tampered regions. However, the bitmaps in [25] are unprotected, and some special tampering could evade the detection using this method, as presented in this section. In contrast, the proposed method is not only able to detect various types of tampering, but also provides the recovery capability of tampered regions.

**Table 6:** Performance comparisons of related methods.

Methods	Generation of authentication code	Detectable		Recoverable
		Type A tampering	Type B tampering	
**[19]**	PRNG	No	No	No
**[20]**	PRNG	No	No	No
**[21]**	PRNG	Partially	No	No
**[22]**	Hash function	Yes	Yes	No
**[23]**	PRNG	Yes	Partially	No
**[24]**	PRNG	No	Yes	No
**[25]**	PRNG	No	Yes	Yes
**Proposed**	Hash function	Yes	Yes	Yes

## Conclusions

In this paper, we propose an efficient authentication method with the capability of recovery for the AMBTC compressed image. The blocks of the AMBTC image are classified into groups according to their similarities. Several copies of the group indices are embedded into the bitmaps of smooth blocks. The dominant portions of the quantization levels, the bitmap, and other required information are hashed to generate the authentication codes. The generated authentication codes are then embedded into the LSBs of the quantization levels. The proposed method successfully detects a variety of tampering, while the tampered blocks are recovered using similar patterns. Experimental results reveal that the proposed method not only outperforms Hu et al.’s and other related methods, but also achieves a very satisfactory marked image quality.
